# A thermostable laccase from *Thermus* sp. 2.9 and its potential for delignification of *Eucalyptus* biomass

**DOI:** 10.1186/s13568-019-0748-y

**Published:** 2019-02-12

**Authors:** Laura E. Navas, Fernando D. Martínez, María E. Taverna, Morgan M. Fetherolf, Lindsay D. Eltis, Verónica Nicolau, Diana Estenoz, Eleonora Campos, Graciela B. Benintende, Marcelo F. Berretta

**Affiliations:** 1 Instituto de Microbiología y Zoología Agrícola, Instituto Nacional de Tecnología Agropecuaria (INTA), Nicolás Repetto y De los Reseros s/n., 1686 Hurlingham, Buenos Aires Argentina; 2 Instituto de Desarrollo Tecnológico Para la Industria Química, INTEC (UNL-CONICET), Santa Fe, Argentina; 3 GPol, UTN, Facultad Regional San Francisco, Santa Fe, Argentina; 4 Department of Microbiology & Immunology, The University of British Columbia, Vancouver, BC V6T 1Z3 Canada; 5 Instituto de Biotecnología, Instituto Nacional de Tecnología Agropecuaria (INTA), Hurlingham, Buenos Aires Argentina; 6 Consejo Nacional de Investigaciones Científicas y Técnicas (CONICET), CABA, Argentina

**Keywords:** Delignification, *Eucalyptus globulus* biomass, Redox mediator, Thermostable bacterial laccase, *Thermus*

## Abstract

Laccases are multicopper oxidases that are being studied for their potential application in pretreatment strategies of lignocellulosic feedstocks for bioethanol production. Here, we report the expression and characterization of a predicted laccase (LAC_2.9) from the thermophilic bacterial strain *Thermus* sp. 2.9 and investigate its capacity to delignify lignocellulosic biomass. The purified enzyme displayed a blue color typical of laccases, showed strict copper dependence and retained 80% of its activity after 16 h at 70 °C. At 60 °C, the enzyme oxidized 2,2′-azino-di-(3-ethylbenzthiazoline sulfonate) (ABTS) and 2,6-dimethoxyphenol (DMP) at optimal pH of 5 and 6, respectively. LAC_2.9 had higher substrate specificity (*k*_cat_/*K*_M_) for DMP with a calculated value that accounts for one of the highest reported for laccases. Further, the enzyme oxidized a phenolic lignin model dimer. The incubation of steam-exploded eucalyptus biomass with LAC_2.9 and 1-hydroxybenzotriazole (HBT) as mediator changed the structural properties of the lignocellulose as evidenced by Fourier transform infrared (FTIR) spectroscopy and thermo-gravimetric analysis (TGA). However, this did not increase the yield of sugars released by enzymatic saccharification. In conclusion, LAC_2.9 is a thermostable laccase with potential application in the delignification of lignocellulosic biomass.

**Electronic supplementary material:**

The online version of this article (10.1186/s13568-019-0748-y) contains supplementary material, which is available to authorized users.

## Introduction

Laccases (EC 1.10.3.2, benzenediol:oxygen oxidoreductase) are multicopper oxidases (MCO) that catalyze the oxidation of a variety of phenolic and non-phenolic compounds with the concomitant reduction of molecular oxygen to water (Thurston 2019). These enzymes are widely distributed in nature, occurring in plants, insects, fungi and bacteria (Bertrand et al. 2017). Laccases typically comprise three domains and contain four copper ions arranged in mononuclear and trinuclear clusters: substrate oxidation at the mononuclear site generates electrons that are transferred to the trinuclear site where O_2_ is reduced (Thurston 2019). Due to their low substrate specificity and high oxidative activity, they have been used in a broad spectrum of applications including pulp bleaching in the paper industry and the degradation of dyes in textile effluents (Singh et al. 2011). Since laccases can catalyze the depolymerization of lignin, a recalcitrant polymer, one application of burgeoning interest is in the pretreatment of lignocellulosic biomass for improving enzymatic conversion of cellulose and hemicellulose to fermentable carbohydrates for production of second generation bioethanol (Christopher et al. 2014). The catalytic efficiency of laccases is usually enhanced by small molecules, called mediators, which act as electron carriers between the laccase active site and the substrate structure. Although several natural and synthetic compounds have been identified as mediators, one of the most effective for lignin transformation is 1-hydroxybenzotriazole (HBT) (Christopher et al. 2014; Hilgers et al. 2018).

The most studied and widely used laccases to date are those from wood decay white-rot fungi (Baldrian 2006). However, bacterial laccases possess a variety of attractive characteristics as compared to fungal laccases, especially with respect to pH-range of activity, thermal stability and resistance to chlorides (Chauhan et al. 2017). In addition, laccases from extremophiles have garnered attention for industrial processes that require harsh conditions, with a few reports of laccases isolated from thermophilic bacteria (Al-kahem Al-balawi et al. 2017; Basheer et al. 2017; Fernandes et al. 2007). Indeed, enzymes from different strains of *Thermus thermophilus* have been found to be useful for the bleaching of wheat straw pulp (Zheng et al. 2012) and the decolorization of dyes (Kumari et al. 2018; Liu et al. 2015). We have previously isolated a thermophilic bacterial strain, *Thermus* sp. 2.9, from a hot spring of Argentina (Navas et al. 2014). Among 2719 protein-coding genes predicted from the bacterium’s genome sequence (Navas et al. 2015), we identified a gene designated *lac_2.9* that encodes a protein with amino acid sequence similarity to laccases.

In this work, we produced and characterized a recombinant form of LAC_2.9. We investigated the enzyme’s thermostability, specificity for laccase substrates and its ability to transform lignin model compounds. Finally, we studied the ability of LAC_2.9 to modify *Eucalyptus globulus* biomass using Fourier transform infrared (FTIR) spectroscopy and thermogravimetric analysis (TGA), and analyzed the effect of pretreating the biomass with LAC_2.9 on the enzymatic hydrolysis of cellulose. The results are discussed with respect to other laccases.

## Materials and methods

### LAC_2.9 sequence analysis

Amino acid sequences were aligned using ClustalX software (https://www.ebi.ac.uk/Tools/msa/clustalw2/). LAC_2.9 domain prediction and analysis were performed using InterProScan (https://www.ebi.ac.uk/interpro/interproscan.html) and SMART (Simple Modular Architecture Research Tool; http://smart.embl-heidelberg.de/). LipoP (http://www.cbs.dtu.dk/services/LipoP/) was used to predict signal peptidase cleavage sites.

### Cloning, expression and purification of recombinant LAC_2.9

DNA from *Thermus* sp. 2.9 cells was prepared as described previously (Navas et al. 2014). The DNA encoding the mature LAC_2.9 protein with an N-terminal 6xHis tag was amplified from *Thermus* sp. 2.9 genomic DNA by PCR using primers: 5′-aggccttcatatgcatcatcaccatcaccaccaggccccctttcccga-3′ and 5′-tggtggt-gggtctagattagctcacctccagaat-3′ (*Nde*I and *Xba*I restriction sites are underlined). The amplicon was cloned into pJexpress 404 (DNA2.0) using engineered *Nde*I and *Xba*I restriction sites. The recombinant plasmid was transformed into *Escherichia coli* XL-1 Blue MRF’ cells by electroporation, and a recombinant clone was verified by sequencing. Expression of *lac_2.9* was induced by adding IPTG (1 mM final) to the LB medium when the culture reached an OD_600_ of 0.8 and CuSO_4_ was added to a final concentration of 0.5 mM. After a further incubation for 6 h at 37 °C, the cells were harvested and lysed in the presence of 1 mM CuSO_4_. The soluble LAC_2.9 was purified using a Ni–NTA affinity column following the manufacturer’s instructions. The eluted fractions containing the recombinant protein were pooled and equilibrated with PBS either by dialysis or size-exclusion chromatography. Protein samples were analyzed using 12% SDS-PAGE and stored at − 70 °C.

### Protein characterization

Protein concentrations were determined using the Pierce™ BCA Protein Assay Kit (Thermo Fisher Scientific, Waltham, MA, USA) with bovine serum albumin as a standard. The copper content of LAC_2.9 was determined using 2,2′-bicinchoninic acid (BCA) after reduction of copper ions released from the holoenzyme (Brenner and Harris 1995).

### Kinetic assays

The activity of LAC_2.9 was detected colorimetrically through the oxidation of ABTS (2,2′-azino-di-[3-ethylbenzthiazoline sulfonate]) and DMP (2,6-dimethoxyphenol) by measuring the absorbance at 436 nm (ε = 36,000 M^−1^ cm^−1^) and 468 nm (ε = 49,600 M^−1^ cm^−1^), respectively. Standard assays were performed at 60 °C and contained 1 mM CuSO_4_ and either 3 mM ABTS (20 mM sodium acetate, pH 5) or 1 mM DMP (20 mM sodium phosphate, pH 6). The dependence of the enzyme activity on copper was evaluated using 0.1–5000 µM CuSO_4_ in the DMP assay. The optimal pH for each substrate was evaluated over a range between pH 3 and 8 using 20 mM sodium acetate (pH 3–5) and 20 mM sodium phosphate (pH 6–8) buffers. The thermostability of the enzyme was analyzed by measuring the residual activity on ABTS at pH 5, after incubating the enzyme at 60, 70 and 80 °C for 2, 6 and 16 h. The laccase activity was calculated as the enzymatic units (U) per ml, with 1 U being the amount of enzyme needed to oxidize 1 μmol of ABTS in 1 min. The effect of 1 mM Mg^2+^, Mn^2+^, Zn^2+^, Ca^2+^, sodium dodecyl sulfate (SDS), ethylenediaminetetraacetic acid (EDTA), dithiothreitol (DTT) and 1–100 mM NaCl on LAC_2.9 activity was assayed by incubating the enzyme with each effector in the reaction mix prior to the addition of ABTS, after which the reaction was carried out as described previously. The enzyme activity in a control reaction without effector was set to 100%. All measurements were carried out in triplicate.

Steady-state kinetic parameters were evaluated at 60 °C using different concentrations of ABTS (50–6000 μM) in 20 mM sodium acetate, pH 5 and DMP (0.2–1500 μM) at 20 mM sodium phosphate, pH 6. Reaction mixtures also contained 1 mM CuSO_4_. Steady-state rate equations were fitted to data using the least-squares and dynamic-weighting options of the program LEONORA (Cornish-Bowden 1995). Curves of initial velocity vs. substrate concentration were plotted using Origin software (Deschenes and Vanden Bout 2000).

### Transformation of lignin model compounds

The ability of LAC_2.9 to transform guaiacylglycerol-β-guaiacyl ether and veratrylglycerol-β-guaiacyl ether was performed essentially as described by Brown et al. (2012). Briefly, 1 mM of lignin model compound (LMC) was incubated with 0.2 μM LAC_2.9 in 20 mM sodium phosphate, pH 6 containing 1 mM CuSO_4_. The reactions were incubated at 60 °C with stirring and quenched after 30 min or 6 h by adding acetic acid to 0.5% final concentration. The quenched reaction was centrifuged at 16,000×*g* for 5 min, and the cleared solution was analyzed by reverse-phase HPLC. Samples were analyzed using a Waters 2695 HPLC (Waters, Milford, MA, USA) equipped with a Luna^®^ 5 µm C18(2) column 250 × 4.6 mm (Phenomenex, Torrance, CA, USA) and a UV detector. The column was operated at 0.7 ml min^−1^ and the sample was eluted using a 16.8 ml linear gradient of 1% formic acid in H_2_O to 100% methanol.

### Enzymatic pretreatment of lignocellulosic biomass with LAC_2.9

The lignocellulosic biomass used was the washed, water insoluble (WIS) fraction of *E. globulus* pretreated by steam explosion (conditions: 187.8 °C, 16 min). The relative composition of the steam exploded biomass was: cellulose 62.89 ± 0.81; hemicellulose 4.74 ± 0.08; acid insoluble lignin 31.46 ± 2.29; ashes 0.55. The biomass, and the determination of its composition, were provided by Dr. Mercedes Ballesteros, from Unidad de Biocarburantes, Centro de Investigaciones Energéticas, Medioambientales y Tecnológicas (CIEMAT), Madrid, Spain. Samples of biomass (20 mg) were treated with laccase in 1 ml reactions containing 50 mM sodium acetate, pH 5, 1 mM CuSO_4_, and 1 U of LAC_2.9, in the presence or absence of 1.5 mM HBT or *p*HBA (*para*-hydroxybenzoic acid). In blank treatments laccase was replaced by crude extract of soluble proteins from *E. coli* transformed with the empty expression vector, with or without mediators. Samples were incubated at 120 rpm and 60 °C for 24 h. The solid residue obtained by centrifugation was washed with distilled water and analyzed by FTIR and TGA after being air-dried for 3 days at 50 °C.

Alternatively, the dried samples of biomass treated with LAC_2.9 (and no-enzyme controls) were submitted to an alkaline peroxide extraction in which they were incubated with 1% (w/w) NaOH and 3% (w/w) H_2_O_2_ (with respect to sample dry weight) at 80 °C for 90 min (Babot et al. 2011).

### Analysis of biomass

FTIR spectra were recorded using a Shimadzu FTIR-8201 PC spectrometer (SSI, Kyoto, Japan). Samples were molded into discs of KBr and spectra were recorded from 400 to 2000 cm^−1^ at a resolution of 2 cm^−1^. Hyper IR software was used to analyze spectra. TGA measurements were conducted using a TA2000 Thermal Analysis System (TA Instruments, New Castle, DE, USA) under pyrolytic conditions. Thermograms were recorded from 20 up to 700 °C at rate of 10 °C min^−1^ using ~ 2 mg of sample.

### Saccharification of woody biomass

Samples of biomass treated with LAC_2.9 and extracted wih alkaline peroxide were washed with water and the solids were incubated with a cellulase cocktail (Cellic^®^ CTec2, Novozymes, Bagsværd, Denmark; 0.5 filter paper units per gram of biomass) in 1 ml 50 mM sodium acetate, pH 5.5 for 16 h at 50 °C. The supernatant was recovered by centrifugation and the reducing sugars were quantified using dinitrosalicylic acid (DNS) (Miller 1959). Data from biological and technical replicates were averaged.

## Results

### Sequence analysis of LAC_2.9

Analysis of the *Thermus* sp. 2.9 genome (Navas et al. 2015) revealed a gene encoding a putative MCO (GenBank accession no. KHG66454.1). The 467 amino acid protein, referred to herein as LAC_2.9, shared 98% sequence identity with a predicted MCO from *T. thermophilus* JL-18 (accession no. AFH38224.1). In silico analysis of LAC_2.9 predicted the occurrence of the three conserved domains typical of this class of MCOs as well as four copper-binding motifs, consistent with mononuclear and trinuclear copper centers (Reiss et al. 2013). Comparison with the laccases from *Thermus* characterized to date showed that LAC_2.9 shares 73% sequence identity with *Tth*-laccase of *T. thermophilus* HB27 (Miyazaki 2005) and 97% identity with LacTT from *T*. *thermophilus* SG0.5JP17-16 (Liu et al. 2015) (Additional file 1: Figure S1). A signal peptide cleavage site was predicted between residues 23 and 24 of LAC_2.9, such that the mature polypeptide was predicted to have 444 amino acids and a molecular mass of 49.5 kDa.

### Catalytic properties of recombinant LAC_2.9

**Fig. 1:**
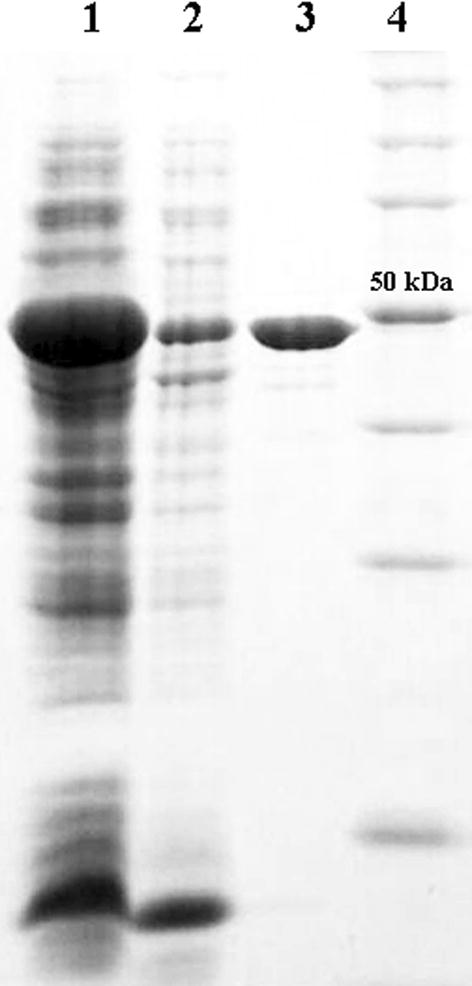
Expression and purification of LAC_2.9. Denaturing gel was loaded with: 1—recombinant *E. coli* culture induced with 1 mM IPTG; 2—recombinant *E. coli* crude extract of soluble proteins; 3—purified LAC_2.9 using Ni–NTA affinity chromatography; and 4—molecular weight standard (Promega, Madison, WI, USA)

To characterize LAC_2.9, the gene encoding the mature protein was cloned and expressed in *E. coli* as an N-terminally His-tagged protein. To maximize specific activity, CuSO_4_ was added to the culture medium at the time of induction and to the lysis buffer. LAC_2.9 was purified by affinity chromatography using Ni–NTA resin. The purified protein exhibited the blue color typical of laccases and had a molecular mass of ~ 49 kDa as judged by SDS-PAGE (Fig. 1). Indeed, the protein has an absorption centered at 621 nm, indicative of a T1 blue copper site. Purified LAC_2.9 had a molar copper content of 4.09 Cu, indicating that the protein was loaded with a full complement of copper.

**Fig. 2:**
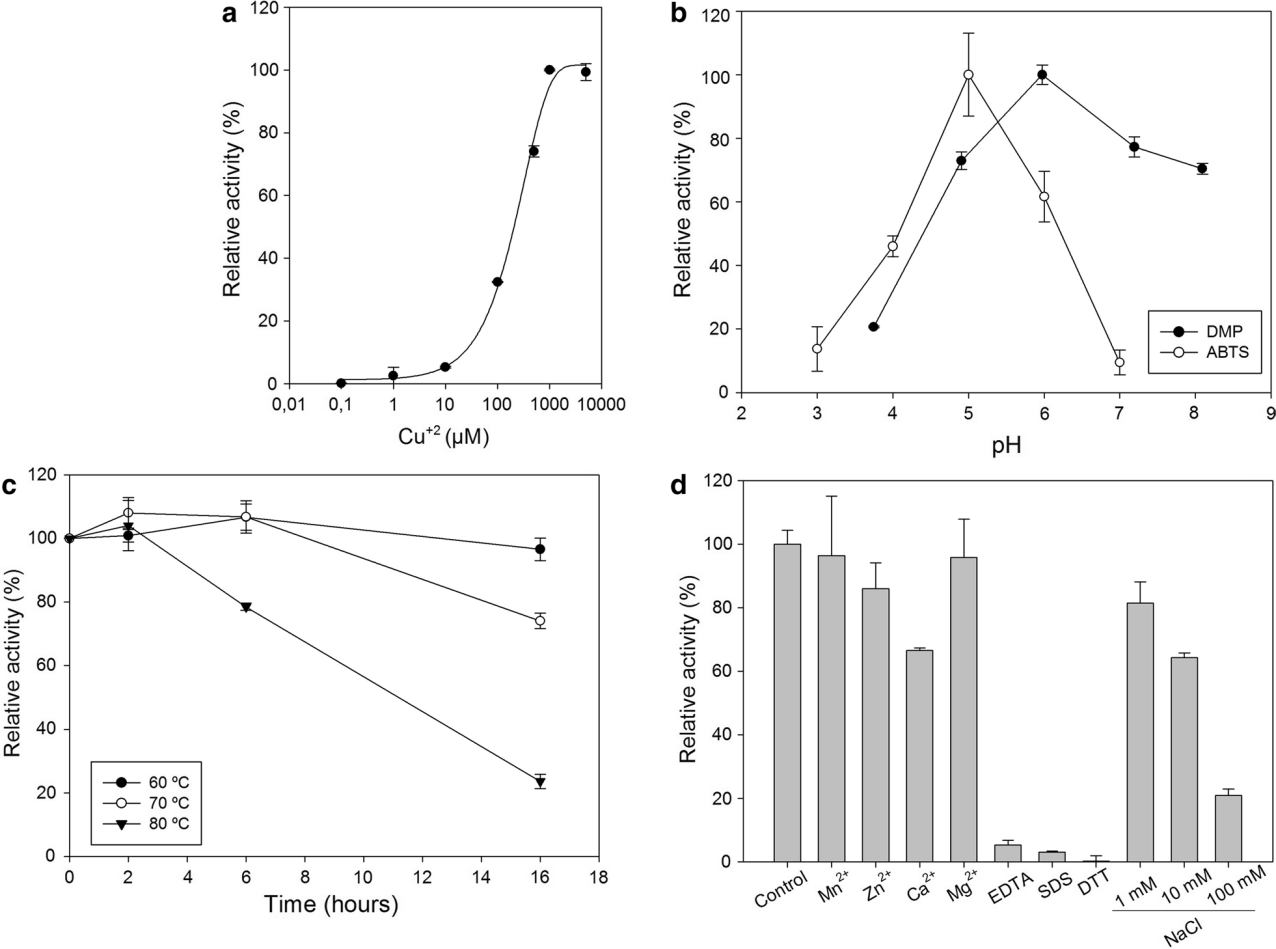
Dependence of LAC_2.9 activity on reaction conditions. **a** Copper dependence of activity; **b** pH dependence of activity; **c** thermal stability profile obtained measuring the residual activity on ABTS at 60 °C, after incubating the enzyme at 60, 70 and 80 °C up to 16 h; **d** effect of metal ions, SDS, EDTA, DTT (1 mM of each) and NaCl (1–100 mM). Assays were carried out in triplicate. The bars indicate standard deviation

Despite containing a full complement of copper, LAC_2.9 requires additional copper ion for activity (Fig. 2a). The dependence of the activity on Cu^2+^ concentration was sigmoidal. Similar behavior has been documented in other bacterial laccases (Basheer et al. 2017; Kim et al. 2015; Miyazaki 2005), with some authors suggesting the existence of an extra copper binding site that is essential for activity. Subsequent kinetic characterization was performed using assay buffers containing 1 mM CuSO_4_.

LAC_2.9 utilized both ABTS and DMP as reducing substrates. However, the pH optimum depended on the substrate: 5.0 and 6.0 for ABTS and DMP, respectively (Fig. 2b). This is similar to what has been reported for other bacterial laccases (Berini et al. 2018). The thermal stability of LAC_2.9 was evaluated by incubating the enzyme for up to 16 h at 60 °C, 70 °C and 80 °C, and measuring the residual activity (Fig. 2c). At 60 °C, LAC_2.9 showed no discernable loss of activity after 16 h. By contrast, 80% of the activity remained after 16 h at 70 °C and after 6 h at 80 °C. Based on this result, subsequent experiments were performed at 60 °C. More generally, this stability indicates that the enzyme is sufficiently robust for industrial applications.

We also investigated the effect of metal ions, EDTA, DTT, SDS detergent and NaCl on the activity of LAC_2.9. Each of these reagents was tested at a concentration of 1 mM (Fig. 2d) and NaCl also at 10 and 100 mM. With respect to metal ions, LAC_2.9 was more resistant than LacTT. In contrast, LacTT was more resistant to SDS and NaCl (Liu et al. 2015). Finally, LAC_2.9 was sensitive to EDTA, a common characteristic of laccases that depend on additional Cu^2+^ for activity (Kim et al. 2015; Miyazaki 2005).

### Steady-state kinetic parameters

The steady-state kinetic parameters of LAC_2.9 were evaluated for ABTS and DMP at pH 5.0 and 6.0, respectively. The enzyme followed Michaelis–Menten behavior for both substrates (Additional file 1: Figure S2). LAC_2.9 had > 50-fold higher substrate specificity (*k*_cat_*/K*_m_) for DMP than for ABTS at their respective optimal pH (Table 1). Furthermore, this specificity for DMP was > threefold higher than that reported for *T. thermophilus* laccases (Table 1), and in general, for most bacterial laccases, although the specificity of bacterial laccases against DMP is generally lower than that of fungal laccases (Additional file 1: Table S1). By contrast, the specificity of LAC_2.9 for ABTS is among the lowest reported for laccases from *T. thermophilus* strains. Finally, despite the high amino acid sequence identity between LAC_2.9 and LacTT from *T. thermophilus* SG0.5JP17-16 (Liu et al. 2015), their respective substrate specificities are quite different, with LacTT having a higher substrate specificity for ABTS than for DMP.

**Table 1:** Comparison of the steady-state kinetic parameters of LAC_2.9 with those of laccases from *Thermus thermophilus* strains

	ABTS			DMP		
	*K*_*m*_ (mM)	*k*_*cat*_ (s^−1^)	*k*_*cat*_*/K*_*m*_ (mM^−1^ s^−1^)	*K*_*m*_ (mM)	*k*_*cat*_ (s^−1^)	*k*_*cat*_*/K*_*m*_ (mM^−1^ s^−1^)
LAC_2.9^a^	0.59 ± 0.08	1.00 ± 0.04	1.7 ± 0.2	0.119 ± 0.006	11.6 ± 0.3	97 ± 3
Tth-laccase^b^	0.9	24.6	27.3	na^f^	na	na
Tth-laccase^c^	2.4	4.77	1.98	na	na	na
TtSLAC^d^	0.49	1.48	3.02	0.11	2.9	27
LacTT^e^	0.036 ± 0.003	0.37 ± 0.01	10.13 ± 0.03	0.15 ± 0.01	0.13 ± 0.01	1.00 ± 0.03

^a^Reactions at 60 °C. For ABTS: 20 mM sodium acetate, pH 5. For DMP: 20 mM sodium phosphate, pH 6

^b^Miyazaki (2005). Reactions at 90 °C. Buffer information not available

^c^Kumari et al. (2018). Reactions at 60 °C. Buffer Britton and Robinson, pH 4.5

^d^Kim et al. (2015). Reactions at 75 °C. For ABTS: 50 mM sodium acetate, pH 4.5. For DMP: buffer information not available

^e^Liu et al. (2015). Reactions at 90 °C. For ABTS: buffer Britton and Robinson, pH 4.5. For DMP: buffer Britton and Robinson, pH 8

^f^Not available

### Reactivity towards lignin model compounds

**Fig. 3:**
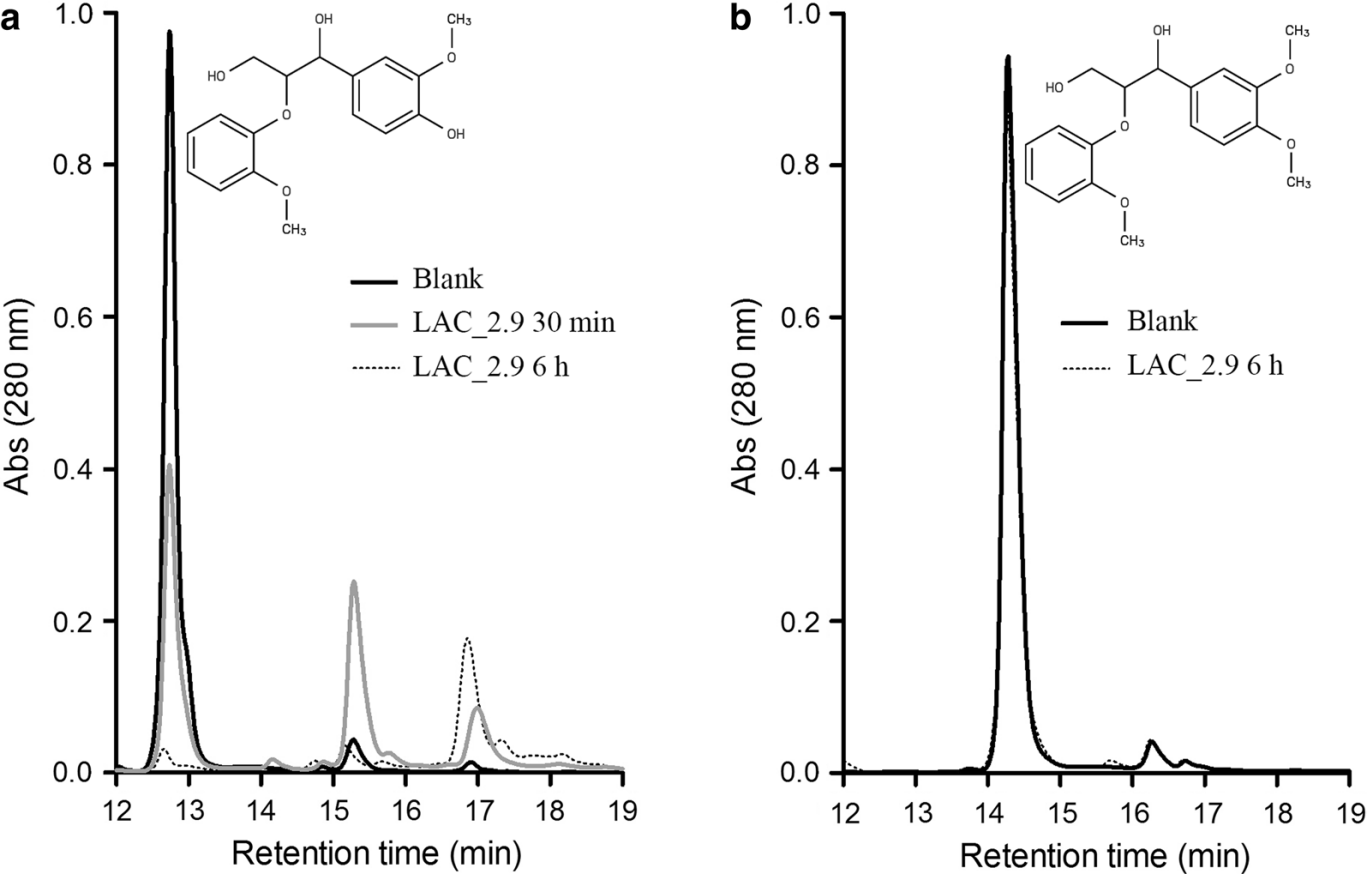
Reactivity of LAC_2.9 with β-*O*-4 biaryl ethers. HPLC traces of reactions performed with **a** guaiacylglycerol-β-guaiacyl ether and **b** veratrylglycerol-β-guaiacyl ether. Reactions were incubated at 60 °C for 30 min and/or 6 h

Due to the complexity of native lignin, model compounds have been used to study the ability of laccases and peroxidases to transform lignin structures. Since β-*O*-4 linkages represent the majority of linkages between lignin subunits, we tested the reactivity of LAC_2.9 towards guaiacylglycerol-β-guaiacyl ether (GGE) and veratrylglycerol-β-guaiacyl ether (VBG), which are phenolic and non-phenolic ethers, respectively. Upon incubation with LAC_2.9, the peak corresponding to GGE decreased with time, and two new peaks appeared later (Fig. 3). In contrast, the peak of VBG remained unchanged after treatment. These results indicate that LAC_2.9 is able to transform the phenolic substrate in the absence of mediators. The retention time of the GGE oxidation products suggests that they are oligomerization products as reported by Hilgers et al. (2018). Overall, it appears that under our experimental conditions, LAC_2.9 catalyzes the oxidative coupling of GGE units, and not the cleavage of the β-*O*-4 bond.

### Pretreatment with LAC_2.9 and saccharification of woody biomass

**Fig. 4:**
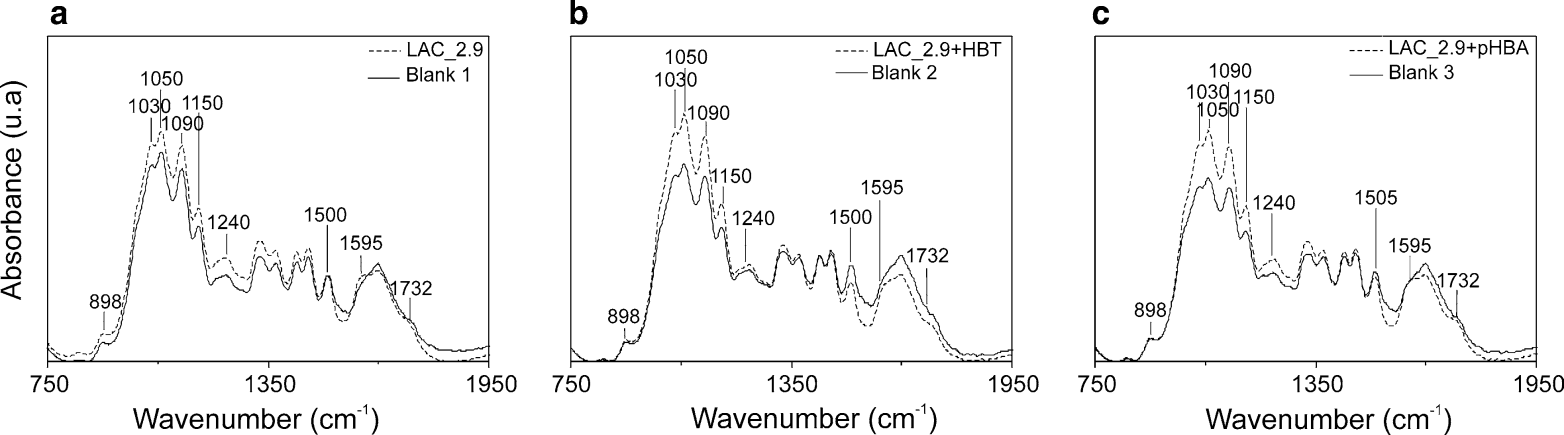
FTIR spectra of LAC_2.9-treated eucalyptus biomass. Samples were treated with: **a** LAC_2.9; **b** LAC_2.9 and HBT; and **c** LAC_2.9 with *p*HBA. Dotted lines represent spectra of enzyme-treated samples. Solid lines represent spectra of equivalent no-enzyme controls treated with cellular extract of *E. coli*

To evaluate the potential of LAC_2.9 for processing lignocellulose, the enzyme was incubated with steam-exploded biomass from eucalyptus at 60 °C for 24 h in the absence and presence of redox mediators. The enzyme-treated biomass was then analyzed using FTIR spectroscopy to infer structural changes in each of the lignin, cellulose and hemicellulose components using band assignments described by Rodrigues et al. (1998) and Avanthi and Banerjee (2016). Inspection of the spectra revealed that incubation with LAC_2.9 in the absence of mediator induced no differences in the bands at 1500/1505 and 1595 cm^−1^ (C=C stretching vibration), corresponding to lignin’s aromatic backbone, and the band at 1732 cm^−1^, corresponding to a C=O stretching vibration associated with a lignin–hemicellulose linkage (Fig. 4a). However, in samples incubated with the laccase in presence of HBT, a relative decrease in the intensity of the bands at 1500 and 1732 cm^−1^ was observed (Fig. 4b). In all LAC_2.9-treated samples, the bands arising from cellulose (898 cm^−1^, 1030 cm^−1^, 1050 cm^−1^, 1090 cm^−1^, 1150 cm^−1^, and 1240 cm^−1^) were slightly more intense as compared to no-enzyme controls. This increase in intensity was more pronounced when mediators were also included, especially for the bands at 1030 cm^−1^, 1050 cm^−1^ and 1090 cm^−1^ (Fig. 4b, c). Similar changes in FTIR spectra have been associated with the delignification of lignocellulosic biomass (Avanthi and Banerjee 2016).

**Fig. 5:**
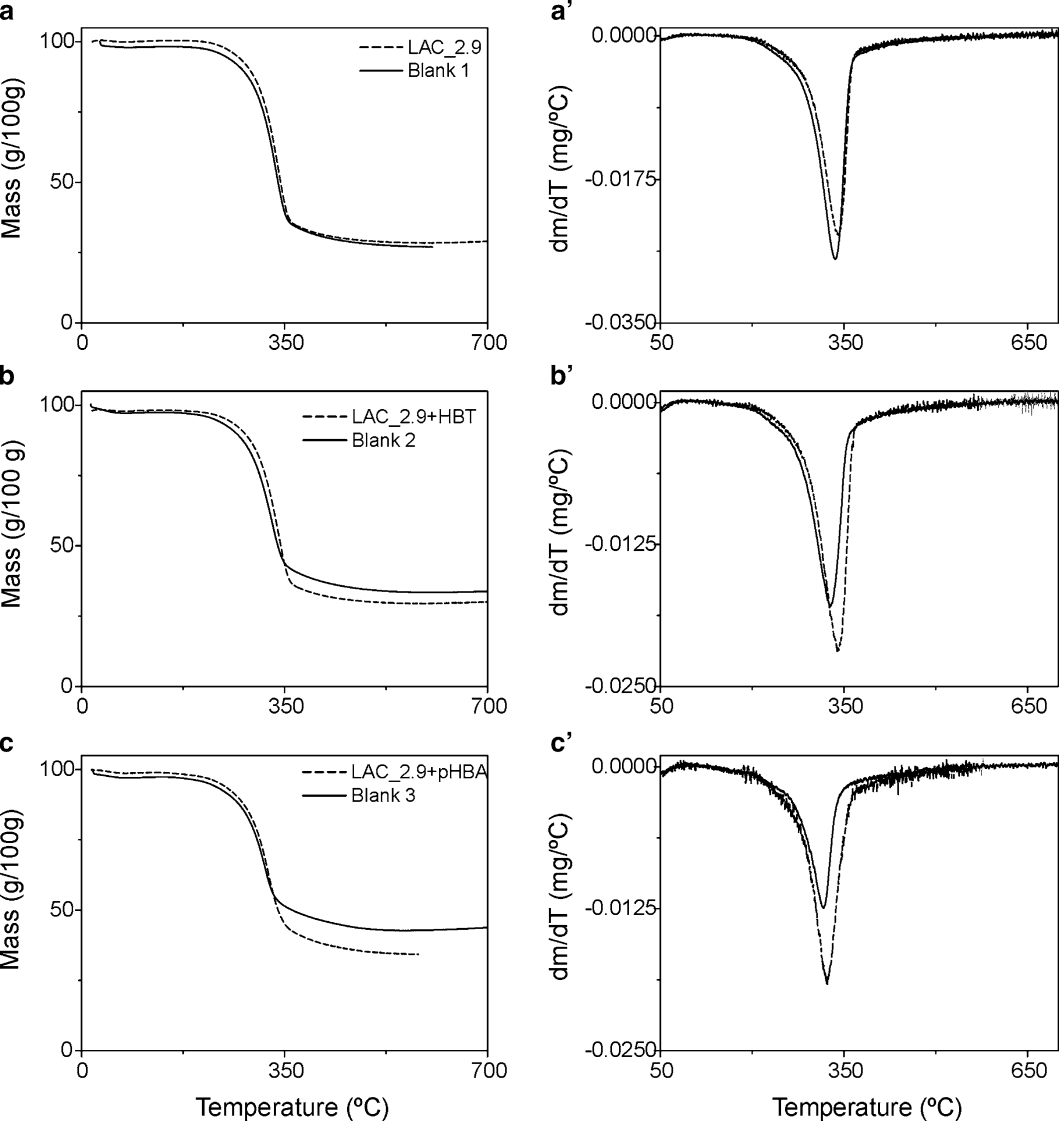
TGA analysis of LAC_2.9-treated eucalyptus biomass. Thermograms (left) and the first derivative curves (right) of biomass treated with (dotted line) and without (solid line) laccase using: **a**, **a**’ LAC_2.9; **b**, **b**’ LAC_2.9 with HBT redox mediator; **c**, **c**’ LAC_2.9 with *p*HBA redox mediator. In no-enzyme controls, LAC_2.9 was replaced with cellular extract of *E. coli*

To further evaluate the effect of LAC_2.9 on woody biomass, we investigated the thermal degradation profile of the enzyme-treated samples. Under pyrolytic conditions in an inert atmosphere, the eucalyptus exhibited a single mass loss step due primarily to the volatilization of cellulose and hemicelluloses (Fig. 5), leaving a carbonaceous residue (char) which mainly results from the lignin (Vila et al. 2011). Inspection of the thermogram (Fig. 5a) indicates that LAC_2.9-treatment did not affect the percentage of char (28%) as compared to a no-enzyme control. However, the addition of mediators with the enzyme resulted in a residual mass decrease of 4 percentage points for HBT (Fig. 5b) and 8 percentage points for *p*HBA (Fig. 5c), which suggests the partial removal of lignin. As illustrated by the first derivative of the thermograms (Fig. 5a′, b′ and c′), all LAC_2.9 treatments increased the temperature of thermal decomposition in comparison to no-enzyme controls. This is observed as a shift to higher temperatures for the maximum rate of weight loss: 6 °C for laccase alone, 11 °C for laccase with HBT and 7 °C for laccase with *p*HBA. Additionally, the ratio of maximum rates of weight loss in laccase treatments versus controls is comparatively higher in treatments with mediators. Collectively the modifications observed in the biomass after the treatment with the laccase-mediator systems suggest degradation of lignin and hemicelluloses adhered to cellulose. These results mirror what has been reported for the treatment of cypress biomass with a fungal laccase (Moniruzzaman et al. 2015) and for the laccase-mediator treatment of flax pulp (Vila et al. 2011). Based on X-ray diffraction, Avanthi and Banerjee (2016) further reported that treatment of lignocellulosic biomass with laccase results in a more ordered arrangement of cellulose after.

To evaluate whether the LAC_2.9-induced changes in the woody biomass correlated with an improvement of the saccharification of residual cellulose and hemicellulose fractions, samples were extracted once with alkaline hydrogen peroxide and then hydrolyzed with a cellulase cocktail, and the release of reducing sugars was measured. None of the laccase treatments, with or without mediators, resulted in significant differences in the yield of released sugars (Additional file 1: Table S2). This suggests that in our experimental conditions, the changes detected in the biomass by spectroscopic and thermal analyses after laccase and laccase-HBT system treatments did not render cellulose and hemicellulose more accessible to enzymatic attack.

## Discussion

Laccases have gained interest for application in several industrial fields and enzymatic bioremediation (Singh et al. 2011). Due to their capacity to modify lignin they have potential for being integrated in emerging biorefineries for the production of lignocellulose-derived ethanol and the conversion of lignin into products of higher value (Fillat et al. 2017). Enzymes best suited for such applications should be highly stable at adverse operational conditions. In this sense, bacterial laccases have comparative advantages with respect to fungal laccases (Chauhan et al. 2017). Since the production of these enzymes from their native sources is usually low, their expression in heterologous hosts may provide the required level of production to fulfill the commercial demand.

In this work we have cloned and expressed in *E. coli* LAC_2.9, a laccase from the thermophilic bacterial strain *Thermus* sp. 2.9. We studied the catalytic properties of the enzyme towards typical laccase substrates and assessed its ability to enhance the enzymatic saccharification of woody biomass.

The amino acid sequence of LAC_2.9 reveals the presence of 3 domains and shares high identity with two previously characterized enzymes from different strains of *T. thermophilus*, *Tth*-laccase (Miyazaki 2005) and LacTT (Liu et al. 2015). Another laccase from *T. thermophilus* was characterized by Kim et al. (2015); this enzyme is a low molecular weight protein, not related in sequence to the previous ones, and has been assigned to a different protein family (Pfam Cu-oxidase_4).

A key factor of successful expression of laccases in heterologous systems consists in obtaining a soluble, properly folded holoenzyme, with a full complement of copper. To this end, it is required to supplement the culture medium with Cu. For *E. coli* it is known that intracellular Cu accumulates at higher concentration during oxygen-limited growth (Outten et al. 2001); consequently, under this growing condition, a fully active holoenzyme may be produced, but at the cost of a lowered productivity. Alternatively, as in the case of CotA laccase from *Bacillus subtilis*, the holoenzyme may also be reconstituted in vitro (Durao et al. 2008). Though, the activity of fully Cu-loaded CotA has been shown to vary depending on whether Cu was incorporated in vivo or in vitro. To produce LAC_2.9, we supplemented the culture medium with Cu at the time of induction and continued growing *E. coli* under aerobic conditions. Subsequently, cells were lysed in buffer also containing Cu and the soluble, His-tagged protein was purified by Ni–NTA affinity chromatography. With this method we obtained LAC_2.9 with a Cu content of c.a. 4 mol Cu per mol protein, as expected for a complete monomeric holoenzyme. We found that LAC_2.9 also required additional Cu^2+^ in the reaction for activity. This requirement was observed in other bacterial MCOs, including LAC_2.9 homologs of *T. thermophilus* (Miyazaki 2005; Liu et al. 2015), and has been associated with the presence of a methionine-rich sequence. Structural analysis by Roberts et al. (2003) revealed the methionines are involved in binding a labile copper ion in CueO, an MCO from *E. coli*. For the McoA enzyme of *Aquifex aeolicus* it was suggested that the binding of Cu^2+^ to the methionine-rich region leads to steric changes that facilitates the oxidation of organic substrates (Fernandes et al. 2007).

LAC_2.9 exhibited a remarkable high substrate specificity (*k*_cat_*/K*_m_) for the phenolic substrate DMP, only comparable to the highest values reported for CotA laccase in a study aimed at identifying experimental conditions conducive to obtaining the fully active recombinant enzyme (Durao et al. 2008). According to this specificity, LAC_2.9 also showed reactivity towards a phenolic lignin model compound (LMC) but not towards a non-phenolic LMC. This reaction appeared to result in the oligomerization of the LMC units. Similar results were reported for experiments using fungal laccases (Hilgers et al. 2018; Ramalingam et al. 2017).

The *Tth*-laccase from *T. thermophilus* has been applied to the bleaching of wheat straw pulp with the result that pulp brightness was increased and the residual lignin content was decreased (Zheng et al. 2012). In the present work, we studied the ability of LAC_2.9 to change the structural properties and to improve the hydrolysability of steam-exploded eucalyptus biomass. Using FTIR spectroscopy we found differences in the spectra of samples treated with LAC_2.9-HBT at bands corresponding to functional groups associated with lignin. These modifications may have arisen from delocalization of electrons, induced by the formation of active radicals from the phenolic subunits of lignin, as one of several possible reactive events (Munk et al. 2014). Additionally, TGA data suggested a slight decrease in the relative lignin content of the biomass after being treated with LAC_2.9-HBT. In spite of these results, no increase in the enzymatic conversion to sugar was obtained after saccharification of LAC_2.9-HBT-pretreated biomass with a cellulose cocktail. This result was similar to that obtained by De La Torre et al. (2017) when treating steam-exploded wheat straw with a fungal laccase, although they found a bacterial laccase to improve the hydrolysability of the same material. Other authors have already observed and studied cases where the amount of sugar released was not improved after a laccase treatment. Yu et al. (2014) tested different enzymatic hydrolysis strategies combined with a laccase pretreatment and observed inhibition by active laccases and oxidized HBT. Oliva-Taravilla et al. (2015, 2016) explained the inhibition of the hydrolytic enzymes either by the phenol oligomers formed by laccases or by the adsorption of the enzymes into lignin present in the biomass sample.

Our results warrant further studies to determine the modifications that LAC_2.9 may produce in different lignocellulosic feedstocks and to realize its potential for improving the efficiency of processes for their conversion into added value products.

## Additional file

**Additional file 1.** Additional tables and figures.
