# Supplementary material for: A thermostable laccase from Thermus sp. 2.9 and its potential for delignification of Eucalyptus biomass
Source: AMB Express. 2019 Feb 12;9:24. doi: 10.1186/s13568-019-0748-y (PMC6372703; doi:10.1186/s13568-019-0748-y)
Supplement: Supplementary file 1 — Additional file 1. Additional tables and figures. [file 13568_2019_748_MOESM1_ESM.pdf]

Additional file 1

A thermostable laccase from *Thermus* sp. 2.9 and its potential for delignification of *Eucalyptus* biomass

Laura E. Navas [1,6], Fernando D. Martínez [1], María E. Taverna [2,3,6], Morgan M. Fetherolf [4], Lindsay D. Eltis [4], Verónica Nicolau [3,6], Diana Estenoz [2,6], Eleonora Campos [5,6], Graciela B. Benintende [1], Marcelo F. Berretta [1,6]

[1] Instituto Nacional de Tecnología Agropecuaria (INTA). Instituto de Microbiología y Zoología Agrícola. Buenos Aires, Argentina.

[2] Instituto de Desarrollo Tecnológico para la Industria Química, INTEC (UNL-CONICET). Santa Fe, Argentina.

[3] GPol, UTN, Facultad Regional San Francisco. Santa Fe, Argentina.

[4] Department of Microbiology & Immunology, The University of British Columbia, Vancouver, BC, V6T 1Z3 Canada.

[5] Instituto Nacional de Tecnología Agropecuaria (INTA). Instituto de Biotecnología. Buenos Aires, Argentina.

[6] Consejo Nacional de Investigaciones Científicas y Técnicas (CONICET). CABA, Argentina.

Corresponding author

Marcelo Berretta

Instituto de Microbiología y Zoología Agrícola, Instituto Nacional de Tecnología Agropecuaria (INTA)

Nicolás Repetto y De los Reseros s/n, 1686 Hurlingham, Buenos Aires, Argentina

E-mail: berretta.marcelo@inta.gob.ar

Phone: +54 11 4621-0799/0125/1684/1448/3207/8496 int 3756

ORCID ID: 0000-0002-6065-4626

Table S1. Substrate specificity (*kcat/Km*) for DMP of bacterial and fungal laccases

Organism	k_{cat}/K_m (mM^{-1} s^{-1})	Reference
**Bacterium**		
Bacillus licheniformis	493,8	Koschorreck et al., 2008
Bacillus subtilis	134,2	Durão et al., 2008
Thermus sp. 2.9	97	This work
Bacillus tequilensis	87	Sondhi et al., 2014
Bacillus sp. HR03	56,6	Mohammadian et al., 2010
Bacillus coagulans	27	Ihssen et al., 2015
Thermus thermophilus HJ6	27	Kim et al., 2015
Bacillus pumilus	16	Reiss et al., 2011
Bacillus clausii	7,6	Ihssen et al., 2015
Metagenome	6,4	Ausec et al., 2017
Streptomyces coelicolor	3,5	Sherif et al., 2013
Thermus thermophilus SG0.5JP17-16	1	Liu et al., 2015
Streptomyces ipomoea CECT 3341	0,98	Molina-Guijarro et al., 2009
Campylobacter jejuni CGUG11284	0,05	Silva et al., 2012
**Fungus**		
Trametes pubescens	5555,5	Galhaup et al., 2002
Pycnoporus sanguineus	2068,4	Ramírez-Cavazos et al., 2014
Meripilus giganteus	1259	Schmidt et al., 2012
Pycnoporus sanguineus	812,2	Ramírez-Cavazos et al., 2014
Cerrena unicolor	792	Michniewicz et al., 2006
Cerrena unicolor	661	Michniewicz et al., 2006
Lentinus sp. nLcc4	325,2	Maestre-Reyna et al., 2015
Cerrena sp. WR1	278,1	Chen et al., 2012
Trametes trogii BAFC 463	160	Campos et al., 2016
Cerrena sp. RSD1	140,3	Wu et al., 2018
Phoma sp	16	Junghanns et al., 2009
Agaricus blazei	14	Ulrich et al., 2005

References:

Ausec L, Berini F, Casciello C, Cretoiu MS, van Elsas JD, Marinelli F, Mandic-Mulec I (2017) The first acidobacterial laccase-like multicopper oxidase revealed by metagenomics shows high salt and thermo-tolerance. Appl Microbiol Biotechnol 101:6261-6276. doi: 10.1007/s00253-017-8345-y

Campos P, Levin L, Wirth S (2016) Heterologous production, characterization and dye decolorization ability of a novel thermostable laccase isoenzyme from *Trametes trogii* BAFC 463. Process Biochem 51:895-903. doi: 10.1016/j.procbio.2016.03.015

Chen SC, Wu PH, Su YC, Wen TN, Wei YS, Wang NC, Hsu CA, Wang AH, Shyur LF (2012) Biochemical characterization of a novel laccase from the basidiomycete fungus *Cerrena* sp. WR1. Protein Eng Des Sel 25:761-769. doi: 10.1093/protein/gzs082

Durão P, Chen Z, Fernandes AT, Hildebrandt P, Murgida DH, Todorovic S, Pereira MM, Melo EP, Martins LO (2008) Copper incorporation into recombinant CotA laccase from *Bacillus subtilis*: characterization of fully copper loaded enzymes. J Biol Inorg Chem 13:183-193. doi: 10.1007/s00775-007-0312-0

Galhaup C, Goller S, Peterbauer CK, Strauss J, Haltrich D (2002) Characterization of the major laccase isoenzyme from *Trametes pubescens* and regulation of its synthesis by metal ions. Microbiology 148:2159-2169. doi: 10.1099/00221287-148-7-2159

Ihssen J, Reiss R, Luchsinger R, Thöny-Meyer L, Richter M (2015) Biochemical properties and yields of diverse bacterial laccase-like multicopper oxidases expressed in *Escherichia coli*. Sci Rep 5:10465. doi: 10.1038/srep10465

Junghanns C, Pecyna MJ, Böhm D, Jehmlich N, Martin C, von Bergen M, Schauer F, Hofrichter M, Schlosser D (2009) Biochemical and molecular genetic characterisation of a novel laccase produced by the aquatic ascomycete *Phoma* sp. UHH 5-1-03. Appl Microbiol Biotechnol 84:1095-1105. doi: 10.1007/s00253-009-2028-2

Kim HW, Lee SY, Park H, Jeon SJ (2015) Expression, refolding, and characterization of a small laccase from Thermus thermophilus HJ6. Protein Expr Purif 114:37–43. doi: 10.1016/j.pep.2015.06.004

Koschorreck K, Richter SM, Ene AB, Roduner E, Schmid RD, Urlacher VB (2008) Cloning and characterization of a new laccase from *Bacillus licheniformis* catalyzing dimerization of phenolic acids. Appl Microbiol Biotechnol 79:217-224. doi: 10.1007/s00253-008-1417-2

Liu H, Cheng Y, Du B, Tong C, Liang S, Han S, Zheng S, Lin Y (2015) Overexpression of a novel thermostable and chloride-tolerant laccase from *Thermus thermophilus* SG0.5JP17-16 in *Pichia pastoris* and its application in synthetic dye decolorization. PLoS One 10(3):e0119833. doi: 10.1371/journal.pone.0119833

Maestre-Reyna M, Liu WC, Jeng WY, Lee CC, Hsu CA, Wen TN, Wang AH, Shyur LF (2015) Structural and functional roles of glycosylation in fungal laccase from *Lentinus* sp. PLoS One 10(4):e0120601. doi: 10.1371/journal.pone.0120601

Michniewicz A, Ullrich R, Ledakowicz S, Hofrichter M (2006) The white-rot fungus *Cerrena unicolor* strain 137 produces two laccase isoforms with different physico-chemical and catalytic properties. Appl Microbiol Biotechnol 69:682-688. doi: 10.1007/s00253-005-0015-9

Mohammadian M, Fathi-Roudsari M, Mollania N, Badoei-Dalfard A, Khajeh K (2010) Enhanced expression of a recombinant bacterial laccase at low temperature and microaerobic conditions: purification and biochemical characterization. J Ind Microbiol Biotechnol 37:863-869. doi: 10.1007/s10295-010-0734-5

Molina-Guijarro JM, Pérez J, Muñoz-Dorado J, Guillén F, Moya R, Hernández M, Arias ME (2009) Detoxification of azo dyes by a novel pH-versatile , salt-resistant laccase from *Streptomyces ipomoea*. Int Microbiol 12:13-21. doi: 10.2436/20.1501.01.77

Ramírez-Cavazos L, Junghanns C, Ornelas-Soto N, Cárdenas-Chávez D, Hernández-Luna C, Demarche P, Enaud E, García-Morales R, Agathos S, Parra R (2014) Purification and characterization of two thermostable laccases from *Pycnoporus sanguineus* and potential role in degradation of endocrine disrupting chemicals. J Mol Catal B: Enzym 108:32-42. doi: 10.1016/j.molcatb.2014.06.006

Reiss R, Ihssen J, Thöny-Meyer L (2011) *Bacillus pumilus* laccase : a heat stable enzyme with a wide substrate spectrum. BMC Biotechnol 11:19. doi: 10.1186/1472-6750-11-9

Sondhi S, Sharma P, Saini S, Puri N, Gupta N. Purification and characterization of an extracellular, thermo-alkali-stable, metal tolerant laccase from *Bacillus tequilensis* SN4. PLoS One 9(5):e96951. doi: 10.1371/journal.pone.0096951

Sherif M, Waung D, Korbeci B, Mavisakalyan V, Flick R, Brown G, Abou-Zaid M, Yakunin AF, Master ER. (2013) Biochemical studies of the multicopper oxidase (small laccase) from *Streptomyces coelicolor* using bioactive phytochemicals and site-directed mutagenesis. Microb Biotechnol 6:588-597. doi: 10.1111/1751-7915.12068

Silva CS, Durão P, Fillat A, Lindley PF, Martins LO, Bento I (2012) Crystal structure of the multicopper oxidase from the pathogenic bacterium *Campylobacter jejuni* CGUG11284: characterization of a metallo-oxidase. Metallomics 4:37-47. doi: 10.1039/c1mt00156f

Schmidt G, Krings U, Nimtz M, Berger RG (2012) A surfactant tolerant laccase of *Meripilus giganteus*. World J Microbiol Biotechnol 28:1623-1632. doi: 10.1007/s11274-011-0968-z

Ulrich R, Huong LM, Dung NL, Hofrichter M (2005) Laccase from the medicinal mushroom *Agaricus blazei*: production purification and characterization. Appl Microbiol Biotechnol 67:357-363. doi: 10.1007/s00253-004-1861-6

Wu MH, Lee CC, Hsiao AS, Yu SM, Wang AH, Ho TD (2018) Kinetic analysis and structural studies of a high-efficiency laccase from *Cerrena* sp. RSD1. FEBS Open Bio 8:1230-1246. doi: 10.1002/2211-5463.12459

Table S2. Yield of reducing sugars from LAC_2.9-treated steam-exploded hardwood.

Treatment	Recovery of reducing sugars (mg/ml) [a]
Blank1	1.0 ± 0.1
LAC_2.9	0.96 ± 0.05
Blank2	1.07 ± 0.01
LAC_2.9 + HBT	1.03 ± 0.04

[a] Values represent mean ± SD. Comparison of means from Tukey test showed no significant differences at 0.05 level.

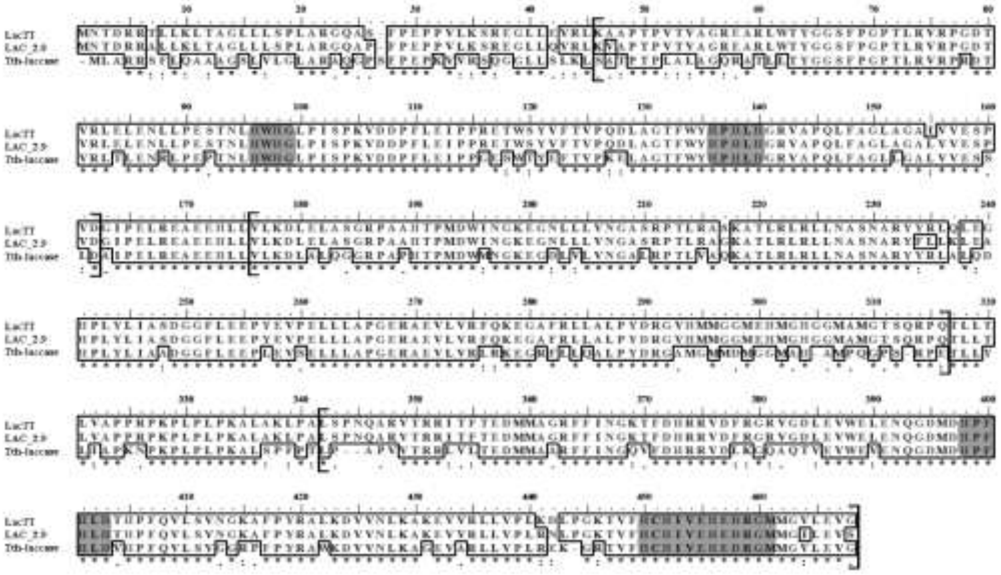

Fig. S1. Alignment of the amino acid sequences of LAC_2.9 and 3-domain lacasses of *Thermus thermophilus* with characterized enzymatic activity, LacTT (YP_005641270) and *Tth*-laccase (AAS81712.1). Predicted domains are delimited by brackets. In LAC_2.9: domain I, residues 45 to 161, MCO type-3 (MCO-3, PFAM accession no. PF07732), domain II, residues 175-312, MCO type-1 (MCO-1, PF00394) and domain III, residues 341-467, MCO type-2 (MCO-2, PF07731). The boxed-in residues comprise positions of identity. The shaded regions indicate the four conserved copper-binding motifs.

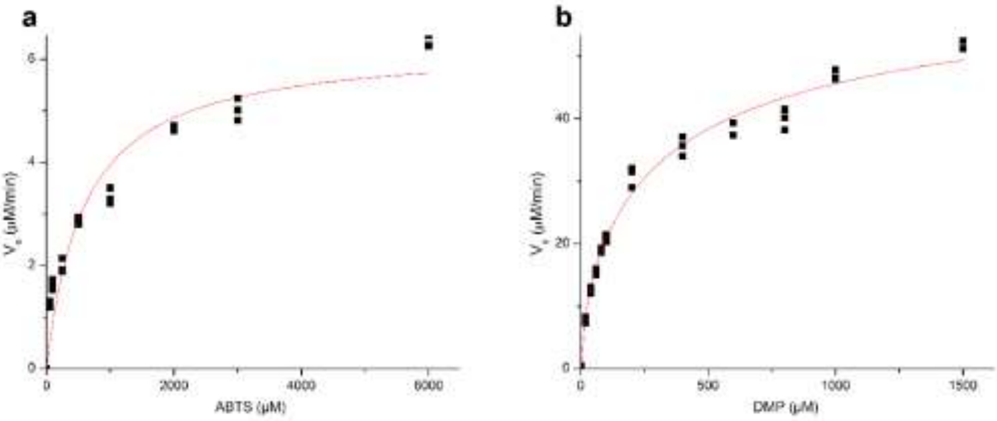

Fig. S2. Steady-state kinetics of LAC_2.9 using (**a**) ABTS and **(b)** DMP. Assays were performed at 60 °C. Reaction mixtures contained 1 mM $CuSO_4$ and either 20 mM sodium acetate, pH 5 for ABTS or 20 mM sodium phosphate, pH 6 for DMP. The experiment was run in triplicate. Curves of initial velocity vs substrate concentration were plotted using Origin software (OriginLab®, Northampton, Massachusetts).
